# Postgraduate Course, Chicago Veterinary College

**Published:** 1903-05

**Authors:** 


### COMMENCEMENT EXERCISES.

### POSTGRADUATE COURSE, CHICAGO VETERINARY COLLEGE.

The following members of this class having complied with the requirements prescribed by the trustees and faculty received their postgraduate diplomas on February 14, 1903:

S. C. Bogart, Chatham, Ontario, Canada (Ontario Veterinary College); O. C. Bradley, Mannington, W. Va. (National Veterinary College); J. F. Fahey, Brockton, Mass. (McGill University, Veterinary Department); C. J. Hackett, Muscatine, Iowa (Western Veterinary College); W. A. Haynes, Jackson, Mich. (Grand Rapids Veterinary College); L. M. Klutz, Clinton, Mo. (Chicago Veterinary College); A. Lames, Dysart, Iowa (Chicago Veterinary College); T. Lambrecht, Montevideo, Minn. (McKillip Veterinary College); R. N. McCarrol, Ft. Collins, Colo. (Western Veterinary College); E. L. Morgenroth, Boltonville, Wis. (Chicago Veterinary

College); J. F. Olweiler, Elizabethtown, Pa. (McKillip Veterinary College); J. M. O'Reilly, Merrill, Wis. (Chicago Veterinary College); J. M. Phillips, St. Louis, Mo. (Chicago Veterinary College); W. B. Scott, Shandon, Ohio (Ontario Veterinary College); W. S. Scott, Oak Park, Ill. (Montreal Veterinary College); G. F. Sheppard, Edinburg, N. D. (Ontario Veterinary College (J. N. Sheppard, Fargo, N. D. (Chicago Veterinary College); F. N. Sherrick, Connellsville, Pa. (Ontario Veterinary College); W. M. Simpson, Malden, Mass. (Montreal Veterinary College); A. Tyler, Elgin, Ill. (Chicago Veterinary College).

